# Extracting default mode network based on graph neural network for resting state fMRI study

**DOI:** 10.3389/fnimg.2022.963125

**Published:** 2022-09-07

**Authors:** Donglin Wang, Qiang Wu, Don Hong

**Affiliations:** Program of Computational and Data Science, Department of Mathematical Sciences, Middle Tennessee State University, Murfreesboro, TN, United States

**Keywords:** default mode network (DMN), graph neural network, graphSAGE, independent component analysis (ICA), rs-fMRI = resting-state fMRI

## Abstract

Functional magnetic resonance imaging (fMRI)-based study of functional connections in the brain has been highlighted by numerous human and animal studies recently, which have provided significant information to explain a wide range of pathological conditions and behavioral characteristics. In this paper, we propose the use of a graph neural network, a deep learning technique called graphSAGE, to investigate resting state fMRI (rs-fMRI) and extract the default mode network (DMN). Comparing typical methods such as seed-based correlation, independent component analysis, and dictionary learning, real data experiment results showed that the graphSAGE is more robust, reliable, and defines a clearer region of interests. In addition, graphSAGE requires fewer and more relaxed assumptions, and considers the single subject analysis and group subjects analysis simultaneously.

## 1. Introduction

Functional connectivity is usually based on two approaches: task-based fMRI analysis and resting-state fMRI analysis. In general, task-based fMRI depends not only on the ability of the subjects to follow the task procedure, but also on the good design of the experiment, especially in clinical applications (Amaro and Barker, 2006; Daliri and Behroozi, 2014; Zhang et al., 2016). In contrast, resting functional magnetic resonance imaging can measure spontaneous fluctuations in the human brain and reflect the relationship among different networks (Biswal et al., 1995; Fox and Raichle, 2007). Therefore, rs-fMRI is applicable to situations in which task-related fMRI may provide insufficient information or sometimes fail to perform (Shimony et al., 2009). In resting state the brain is still constantly active and parts of the brain are connected by its intrinsic connections, and some connections even show a stronger relationship when doing certain tasks. Therefore, it is more reasonable to use resting state to study basic functional connections, especially to identify intrinsic connectivity networks or resting state networks (RSNs) (Greicius et al., 2003; Beckmann et al., 2005; De Luca et al., 2006; Seeley et al., 2007; Canario et al., 2021; Morris et al., 2022). As mentioned in several studies (Beckmann et al., 2005; De Luca et al., 2006; Fox and Raichle, 2007; Smith et al., 2009; Cole et al., 2010), RSNs are located in the gray matter regions of the human brain, these RSNs reflect the core perceptual and cognitive processes of functional brain systems.

There are several common RSNs (Cole et al., 2010; Lee et al., 2013) that are often identified from the resting-state fMRI study. The most basic RSN is the default mode network (DMN) (Greicius et al., 2003; Damoiseaux et al., 2006; Van Den Heuvel M. et al., 2008; van den Heuvel M. P. et al., 2008; Thomas Yeo et al., 2011; Andrews-Hanna et al., 2014; Shafiei et al., 2019) which is active when the human brain is at rest without any external or attention-demanding tasks. Mostly it includes the posterior cingulate cortex (PCC) and the precuneus, the medial prefrontal cortex (MPFC), and the inferior parietal cortex.

The Somatomotor network (SMN) is another network which includes somatosensory and motor regions related to motor tasks (Biswal et al., 1995; Chenji et al., 2016). The visual network (VIN) includes the main part of the occipital cortex, which deals with related visual activity (De Luca et al., 2006; Smith et al., 2009; Power et al., 2011; Thomas Yeo et al., 2011; Hendrikx et al., 2019). The dorsal attention network (DAN), also known as the visuospatial attention network, is used to provide attention orientation and participate in external tasks; it is located primarily in the intraparietal sulcus and the area of connection between the central anterior sulcus and the forehead sulcus (Vincent et al., 2008; Lei et al., 2014; Vossel et al., 2014; Bell and Shine, 2015; Hutton et al., 2019; Shafiei et al., 2019). The Salience Network (SAN) mainly evaluates external stimuli and internal events and helps direct attention by switching to related processing systems.

There are several techniques such as seed-based correlation method, independent component analysis (ICA), and dictionary learning method that identify DMN from rs-fMRI, which will be briefly reviewed in the next section. In Section 3, we propose the use of the graph neural network, a deep leaning technique called graphSAGE (Hamilton et al., 2017) to specifically extract DMN from resting fMRI. In Section 4, data analysis experiments and comparisons are performed based on two real data sets followed by conclusions and final remarks.

## 2. Common methods review

For comparison purposes, we briefly review several typical methods in rs-fMRI data studies, including the seed-based correlation method, independent component analysis (ICA), and the dictionary learning method.

### 2.1. Seed-based correlation

Seed-based correlation (SBC) method was the first method to be used in identifying resting-state networks by Biswal et al. (1995). It calculates the Pearson correlation coefficient between two voxels or region of interests (ROI) from the corresponding two time series, denoted as ***x*** = [*x*_1_, *x*_2_, *x*_3_, ..., *x*_*n*_] and ***y*** = [*y*_1_, *y*_2_, *y*_3_, ..., *y*_*n*_]. The correlation coefficient is calculated as usual


(1) $$\begin{array}{r} r_{xy}=\frac{\sum_{i=1}^{n}\left(x_{i}-\bar{x}\right)\left(y_{i}-\bar{y}\right)}{\sqrt{\sum_{i=1}^{n}{\left(x_{i}-\bar{x}\right)}^{2}\sum_{i=1}^{n}{\left(y_{i}-\bar{y}\right)}^{2}}}, \end{array}$$

where $\bar{x}$ and $\bar{y}$ are the mean values of ***x*** and ***y***, respectively.

Normally, there are approximately one million voxels in one entire brain scan, and the data amount is based on the size of voxels and the scanned brain. Though it is possible to calculate all the correlations between any two voxels, this will cause numerous pairs of comparisons. More often, this method uses a seed or ROI selected earlier as a reference and calculates the correlation with the rest of the brain voxels. A high correlation value means closer connectivity between the two seeds or ROIs. This method has been applied in many research studies and identifies functional connectivity based on rs-fMRI. In Fox et al. (2006), the authors identified a bilateral dorsal attention system, a right-lateralized ventral attention system, and detected a potential mediating function in the prefrontal cortex. In Fox et al. (2005), the authors identified two opposed brain networks based on correlations within each network and anticorrelations between networks. One network is related to task-related activation and the other is related to task-related deactivation. In de Oliveira et al. (2019), the authors used sensorimotor networks as the seed to extract similar RSNs from rs-fMRI with those from finger tapping task fMRI data based on eight healthy volunteers. In addition to a previously selected seed or ROI, this method also needs a threshold to determine the significant voxels with the seed or ROI. The main advantages of SBC are its algorithmic simplicity and straightforward interpretation, which make it play an important role in the study of functional connectivity (FC). The main disadvantage of this method is that all relationships are only between the prior selected seeds or ROIs and the remaining other voxels of the brain. Therefore, the univariate analysis method completely ignores possible relationships between other seeds. In other words, significant information between other voxels is usually ignored. This restricts the detection of more possible networks in FC. Therefore, the choices of seeds, sizes, and positions directly determine the interpretation of the results (Buckner et al., 2008; Cole et al., 2010).

### 2.2. Independent component analysis

Independent component analysis (ICA) is a data-driven technique that is one of the most popular decomposition methods for analyzing fMRI data. Since this technique was first used in the field (McKeown et al., 1998), it has been applied in many studies thus far. ICA assumes that the observed data is a linear combination of sources that are statistically independent (Brown et al., 2001).

The purpose of ICA is to extract independent components based on an optimization technique, such as maximum likelihood estimation (MLE) (Stone, 2004), minimum mutual information between sources (InfoMax) (Bell and Sejnowski, 1995), or maximum non-Gaussianity between sources (FastICA) (Hyvärinen and Oja, 2000).

Because ICA is looking for non-Gaussian sources, most ICA algorithms often use Principal Component Analysis (PCA) (Tharwat, 2016) to remove Gaussian signals in the observed data. The difference between ICA and PCA is that ICA searches statistically independent components with maximum possibility and PCA searches uncorrelated components with an orthogonal property and maximum of variance. Since fMRI is four-dimensional data, it has to be transferred into two-dimensional data when using ICA or PCA. ICA can be one of two types of analysis: spatial ICA and temporal ICA, and different types of analysis determine how to transfer fMRI data. If the spatially independent components are detected, the columns of the transferred matrix are voxels and the rows are the time points; if the temporally independent components are detected, the columns of the transferred matrix are time points and the rows are the voxels. ICA can be divided into spatial ICA and temporal ICA, but spatial ICA is typically used because it can produce as many components as time points (McKeown et al., 1998; Calhoun et al., 2001).

Here is the basic algorithm of the spatial ICA (McKeown et al., 1998; Calhoun et al., 2001). Let **X** be a matrix with dimension *t* × *n* (**X**∈ℝ^*t* × *n*^), where *t* means the number of time points and *n* means the number of voxels. The goal is to solve the following equation to find **W, S**:

(2) $$\begin{array}{r} \text{X}=\text{W}\text{S}, \end{array}$$

where **W** is the matrix of mixing coefficient with dimension *t* × *p*, **S** is the source matrix with dimension *p* × *n*, and the rows of the matrix **S** represent spatially independent components. The basic algorithm of temporal ICA is similar to spatial ICA (McKeown et al., 1998; Calhoun et al., 2001), where **X** is the matrix with dimension *n* × *t* (**X**∈ℝ^*n* × *t*^). The goal is to solve the following equation to find $\hat{\text{W}},Ŝ$:


(3) $$\begin{array}{r} \text{X}=\hat{\text{W}}\hat{\text{S}} \end{array}$$

where $\hat{\text{W}}$ is still the matrix of the mixing coefficient with dimension *n* × *p*, $\hat{\text{S}}$ is the source matrix with dimension *p* × *t*, and the rows of the matrix $\hat{\text{S}}$ represent the temporally independent time courses. Usually, spatial ICA is used more often than temporal ICA.

There are many techniques applied in the ICA. All algorithms fall into one of these three groups: The first is based on the projection pursuit (Stone, 2004), which basically extracts one component at one time. The second is based on infomax (Bell and Sejnowski, 1995) which extracts multiple components at one time in a parallel way. The third is based on the estimate of the maximum likelihood (Stone, 2004), which is a statistical tool to calculate the mixing coefficient matrix to best fit the observed data. There are many practical methods used in the literature, for example, in Beckmann et al. (2005), the authors proposed a probabilistic independent component analysis (PICA), compared with typical noise-free ICA, PICA tried to detect the independent components under additive noise interference. Hyvärinen and Oja (2000), introduced a widely used method called FastICA with kurtosis as a cost function. Beckmann et al. (2009), used multiple subjects and dual regression to do a group comparison. To date, ICA still plays an important role in resting state analysis.

ICA does not provide an order for independent components (ICs), unlike PCA which offers an order for principal components. This property makes it hard to tell which IC is more important. To solve the mixing coefficient **W** and sources **S**, ICA needs prior assumptions. It also becomes tricky to determine the number of ICs, which can cause over-fitting or under-fitting problems.

### 2.3. Dictionary learning

Dictionary learning (DL) is a linear decomposition technique to extract the fundamental components. This technique emphasizes the sparsity between components, unlike ICA which focuses on the independence between components or PCA which focuses on the orthogonal components. The idea of DL is to decompose the observed data into two matrices; a matrix is called the dictionary **D** which is a collection of atoms, and the other is the corresponding spare coefficients **A**. Here is the basic algorithm of dictionary learning (Olshausen and Field, 1996, 1997; Kreutz-Delgado et al., 2003; Mairal et al., 2009; Joneidi, 2019), let **Y** = [*y*_1_, *y*_2_, ..., *y*_*n*_] be the observed data with $y_{i}\in {\mathbb{R}}^{m}$ and **Y**∈ℝ^*m* × *n*^, and the dictionary matrix **D**∈ℝ^*m* × *k*^ (**D** = [*d*_1_, *d*_2_, ..., *d*_*k*_]) with the corresponding sparse coefficient matrix **A**∈ℝ^*k* × *n*^ (**A** = [*a*_1_, *a*_2_, ..., *a*_*n*_]). The number of columns *k* in the dictionary **D** is the number of atoms. The purpose is to find **D** and **A** from the following optimization problem:


(4) $$\begin{array}{r} \underset{\text{D},\text{A}}{\text{Minimize}}\frac{1}{2}{\left||\text{Y}-\text{D}\text{A}|\right|}_{2}^{2}\\ \text{Subject to}{\left||{\text{D}}_{.,i}|\right|}_{2}\leq 1,\forall i=1,\ldots ,k,\\ \;{\left||{\text{A}}_{.,j}|\right|}_{0}\leq C,\forall j=1,\ldots ,n, \end{array}$$

where *C* is the parameter to control the sparsity for each column of **A**.

There are several improved ways to solve the optimization problem. For example, Aharon and Elad (2008), used the stochastic gradient descent technique to solve this problem, and Lee et al. (2007), used the property of duality to solve the optimization problem. A widely used method is the K-SVD method (Aharon et al., 2006), which uses singular value decomposition to generate clustering *K*. It is an iterative process to update the sparse matrix and dictionary matrix alternatively.

## 3. Graph neural network

Brain connectivity patterns from fMRI data are usually classified as statistical dependencies, named functional connectivity or causal interactions, called effective connectivity among various neural units. Investigating the human brain based on connectivity patterns reveals important information about the brain's structural, functional, and causal organization. Graph theory-based methods have recently played a significant role in understanding brain connectivity architecture. A graph neural network (GNN) is a type of deep neural network that is applied to graph-based data. To date, there are many variants of GNN, such as graph convolution networks (GCN), graph attention networks (GAN), graph autoencoders that have been used successfully in many fields including computer vision, recommendation system, and more. Yao et al. (2019), used GCN for text classification in natural language processing. The study by Kosaraju et al. (2019), proposed a new method based on GAN to predict the future trajectories of multiple interacting pedestrians. Pan et al. (2018), proposed a novel graph autoencoder framework to reconstruct the graph structure. Yao et al. (2021), discussed a mutual multi-scale triplet graph convolutional network (MMTGCN) for classification of brain disorders based on fMRI or diffusion MRI data. Yao et al. (2020), proposed a temporal-adaptive graph convolution network (TAGCN) to mine spatial and temporal information using rs-fMRI time series. TCGCN can take advantage of both spatial and temporal information using resting-state functional connectivity (FC) patterns and time-series, and can also the explicitly characterize subject-level specificity of FC patterns.

In graph theory, an undirected and unweighted graph is represented by *G*(*V, E*), where *G* is the graph, *V* is the set of vertices (nodes) and *E* is the set of edges connected between two vertices (Butts, 2008; van den Heuvel M. P. et al., 2008). In fMRI data analysis, a graph *G* represents the network of a brain, *V* represents the set or a subset of voxels or ROIs, and *E* is the set of functional connections or correlations between elements in *V*, especially between the ROIs.

An adjacent matrix **A** with dimension *N* × *N* is used to describe this kind of graph. The entry of **A** is one or zero depending on whether there is an existing edge between two nodes. A matrix *X*∈ℝ^*m* × |*V*|^ often indicates the attributes or features of a graph, where *m* is the number of features of each node (ROI) and |*V*| indicates the number of nodes (ROIs).

GraphSAGE (sample and aggregate) (Hamilton et al., 2017) is an inductive learning technique to extract node embedding. GraphSAGE can be used in unsupervised and supervised learning. Here, specifically for node extraction in fMRI analysis, an unsupervised learning procedure based on GraphSAGE can be implemented as follows:

1) Given a graph *G*(*V, E*) and feature matrix *X*∈ℝ^*m* × |*V*|^; for ∀*v*∈*V*, each *x*_*v*_ has a vector of feature with *m* dimension.
2) Set up a neural network with *K* layers and let $h^{0}=x_{v}$ indicate the embedding of the original node for ∀*v*∈*V*, and this is the feature of the node or the attribute with *m* dimensions originally.
3) For the *k* = 1 layer, and for each v∈V:
  (a) Let N(v) indicate the local neighborhood of nodes of *v*, all information from the neighborhood is aggregated by a function indicated by *f*, then the generalized aggregation is indicated as follows:
    (5) $$\begin{array}{r} h_{N\left(v\right)}^{1}=f_{1}\left(h_{u}^{0}\right),\;u\in N\left(v\right) \end{array}$$
    aggregate function *f* can be one of the three: i) the mean value function $f_{1}=\underset{u\in N\left(v\right)}{\sum }\frac{h_{u}^{0}}{\left|N\left(v\right)\right|}$ which takes the average among all the $h_{u}^{0}$; ii) the pool value function $f_{1}=max\left(W_{pool}h_{u}^{0}\right),\forall u\in N\left(v\right)$ which applies an element-wise max-pooling operation on neighbor set nodes; iii) a long short-term memory (LSTM) function $f_{1}=LSTM\left(h_{u}^{0}\right),u\in \pi \left(v\right)$ which applies LSTM to the random permutation of the neighbor set of nodes.
  (b) After summarizing information from local neighbor sets by the aggregate function, the embedding for each node is in the following form.
    (6) $$\begin{array}{r} h_{v}^{1}=\sigma \left(W^{1}\cdot concatenate\left(h_{v}^{0},\;h_{N\left(v\right)}^{1}\right)\right) \end{array}$$
    where σ is non-linearity function and *W*^1^ is the weight matrix.
4) After all nodes through parts a) and b) from step 3), the node embedding after the first layer is represented below:
  (7) $$\begin{array}{r} h_{v}^{1}=\frac{h_{v}^{1}}{||h_{v}^{1}|{|}_{2}},\;v\in N\left(v\right) \end{array}$$
5) For 2 ≤ *k* ≤ *K* layers step 3) and 4) are repeated. The embedding for each node is in the form below:
  (8) $$\begin{array}{r} h_{N\left(v\right)}^{k}=f_{k}\left(h_{u}^{k-1}\right),\;\forall u\in N\left(v\right)\\ \;h_{v}^{k}=\sigma \left(W^{k}\cdot concatenate\left(h_{v}^{k-1},\;h_{N\left(v\right)}^{k}\right)\right)\\ \;h_{v}^{k}=\frac{h_{v}^{k}}{||h_{v}^{k}|{|}_{2}},\;v\in N\left(v\right) \end{array}$$
  and the common form of aggregate functions is:
  (9) $$\begin{array}{r} f_{k}=\underset{u\in N\left(v\right)}{\sum }\frac{h_{u}^{k-1}}{\left|N\left(v\right)\right|} \end{array}$$
6) The embedding of node after the layer *K* is as follows:
  (10) $$\begin{array}{r} z_{v}=h_{v}^{K},v\in V. \end{array}$$
7) The process is iterated to minimize the loss function as follows:
  (11) $$\begin{array}{r} J\left(z_{u}\right)=-\log\left(\sigma \left(z_{u}^{⊤}z_{v}\right)\right)-Q\cdot {𝔼}_{v_{n}\sim P_{n}\left(v\right)}\log\left(\sigma \left(-z_{u}^{⊤}z_{v_{n}}\right)\right) \end{array}$$

where σ is non-linearity function, *z*_*v*_ is the embedding of neighbor node of *u*, *Q* is the negative samples (non-neighbor node) of *u* and *v*_*n*_ is from negative sample distribution *P*_*n*_(*v*).

As one of the characteristics of GNN, it can provide a learning procedure to identify relationships efficiently between nodes with relatively relaxing restrictions and assumptions. The following applications of the graphSAGE for extracting the default mode network show that the GNN defines ROIs clearer and provides more robust and reliable results in comparing to the traditional methods.

## 4. Data analysis

In this section, two real data sets are used to extract the default mode network, based on the methods mentioned above. A comparison will be made among the methods correspondingly.

Both data sets are obtained from an open shared neuroimage data resource (http://fcon_1000.projects.nitrc.org/fcpClassic/FcpTable.html), namely, the 1000 Functional Connectomes Project.

### 4.1. Case one

The data has 84 subjects including 43 males and 41 females. Their ages range from 7 to 49 years. The repetition time (TR) is 2 s, the number of slices is 39, and the time points are 192.

Data preprocessing is an important step before doing any statistical analysis. There are several software packages that deal with fMRI data preprocessing. For this data set, the DPABI (Yan et al., 2016), a toolbox of Matlab, is used. The first ten time points are removed. The pipeline for preprocessing is as follows; first, time correction is done; this is followed by realignment, co-registration between T1 weighted and functional images; head motion model with Friston 24 parameters motion covariates is used to reduce head motion effect (Friston et al., 1996); nuisance regression with linear trend, average cerebrospinal fluid (CSF) and white matters (WM) as nuisance regressors are to reduce respiratory and cardiac effects; finally normalization to MNI template is performed. The temporal filtering is set in the range of 0.01~ 0.1 Hz and the smoothing at FWHM is equal to 6 mm.

**Figure 1:**
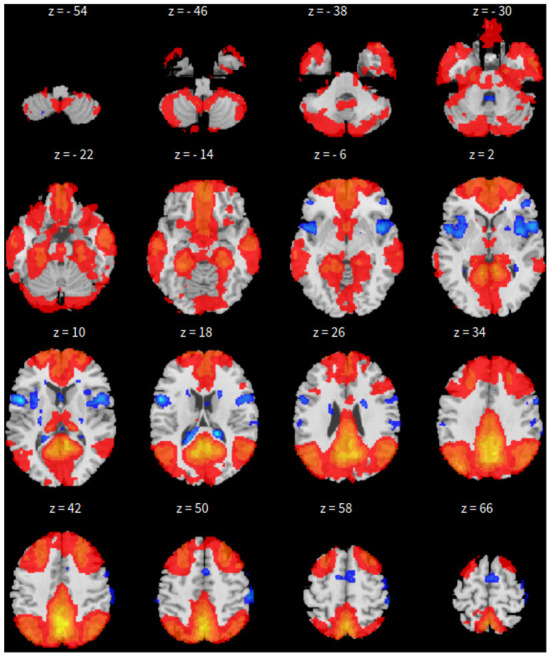
*xy*-plane view of SBC in Case-1.

First, the SBC is performed in the CONN toolbox (Whitfield-Gabrieli and Nieto-Castanon, 2012) in Matlab. The voxel threshold p-uncorrected value is set less than 0.0001 and the familywise error rate (FWER) is set less than 0.01 which attempts to control the probability of false positives. The |*T*| value is larger than 4.09 with degree of freedom 83 for the two-sided test. See Figure 1 for details based on the seed of posterior cingulate cortex (PCC) from the default mode network.

**Figure 2:**
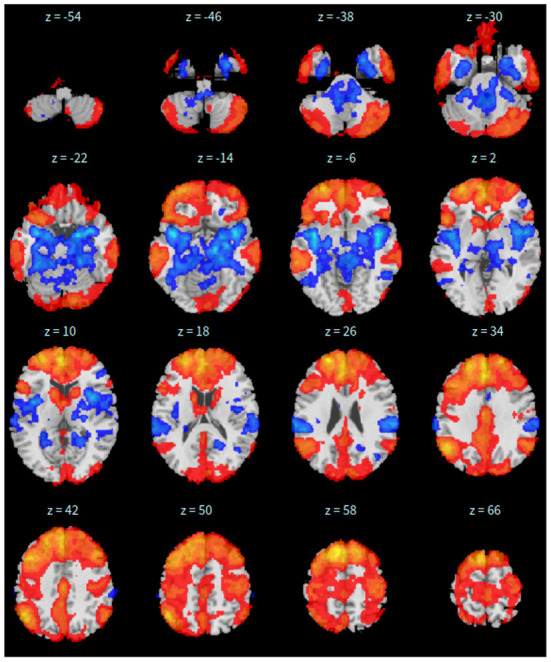
*xy*-plane view of ICA in Case-1.

Second, the group fastICA is also performed in CONN (Whitfield-Gabrieli and Nieto-Castanon, 2012) and the independent components are set to 10. Other parameters are the same as SBC. See Figure 2 for details.

**Figure 3:**
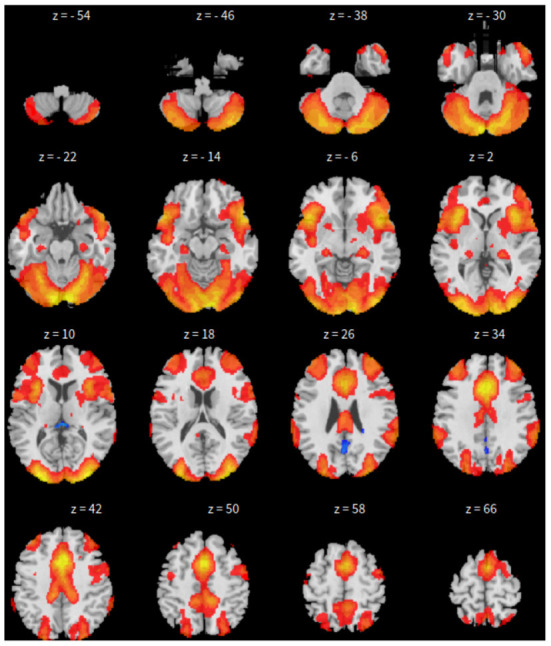
*xy*-plane view for DL in Case-1.

Third, dictionary learning is performed in Python with the Nilearn (Abraham et al., 2014) package. As mentioned in Section 2.3, dictionary learning focuses on sparsity between components. The component is set to 10 and the sparsity parameter is set to 15. See Figure 3 for details.

**Figure 4:**
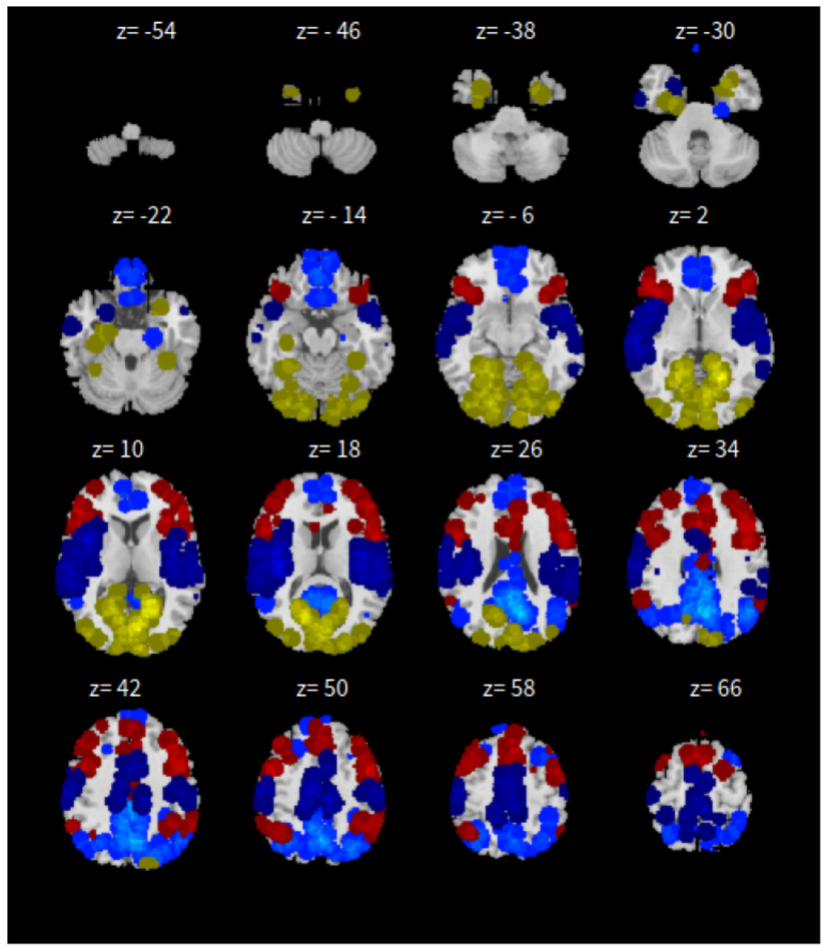
*xy*-plane view for GNN in Case-1.

**Figure 5:**
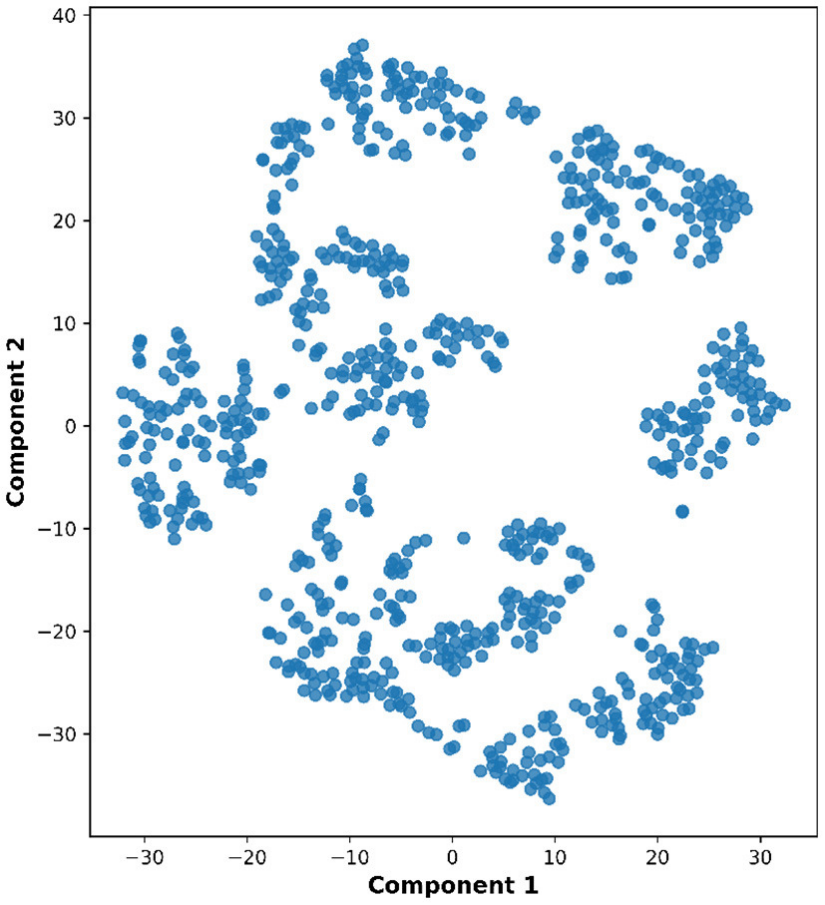
Plot of node embedding in Case-1 with two components.

**Figure 6:**
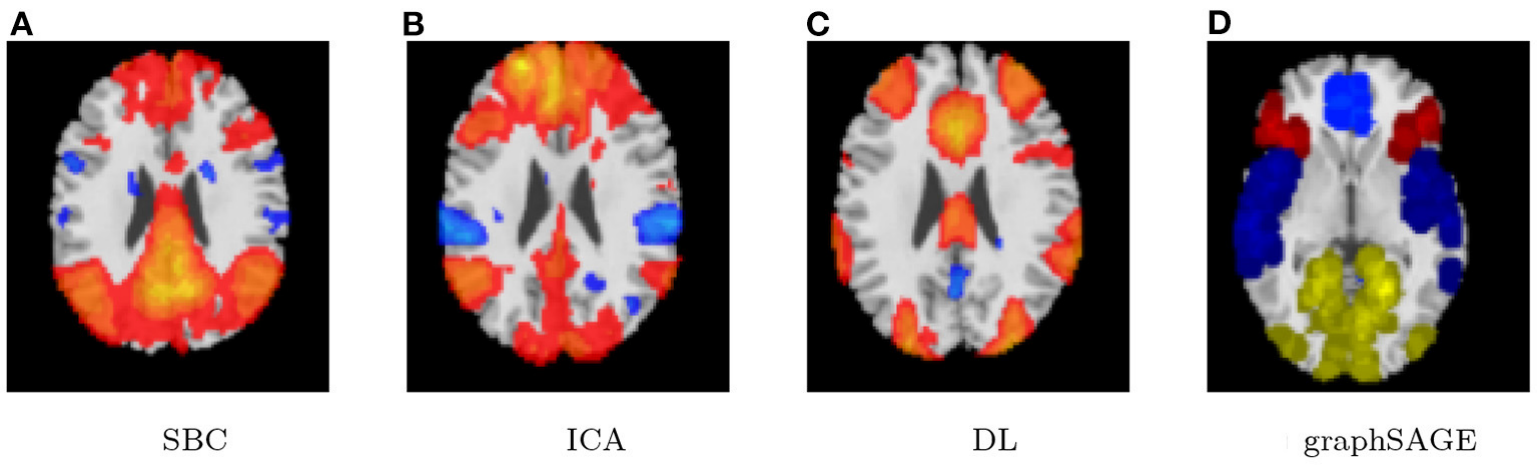
Case-1 DMN result comparison. **(A)** SBC, **(B)** ICA, **(C)** DL, and **(D)** graphSAGE.

Lastly, the graph neural network method GraphSAGE is performed through the package of StellarGraph (Data61, 2018) in Python. See Figure 4 for details. Since the 800 ROIs of the brain are used, there are 800 nodes in the graph and each two makes an edge so that there are a total of 319,600 edges in the graph. For each subject, the correlation between any two ROIs is calculated based on 182 time series data. Each subject has 182 time points, which means that each ROI of each subject has 182 values. Each subject has a correlation matrix with dimension of 800 × 800. There are 84 correlation matrices for 84 subjects. To reduce complexity and noise, all the average correlation values less than 0.1 are replaced by zero, and thus the associated edges are dropped. The correlation for each node is the node feature, which is used in the subsequent analysis. A two-layer graph network is set for analysis. The number of walks for each node *v* is set to 10; the length of walk is 5; batch size is set to 10; 1-hop neighbors node for each node *v* is set to 50 and 2-hop neighbors node is set to 10 for each node *v*; the first layer is set to 200 hidden neurons; the second layer is set to 100 neurons; the learning rate is set to 0.01; epochs is set to 100 and dropout rate is set to 0.15. After analysis each node embedding is represented by a vector with 100 dimensions. To visualize the node embedding, a principal component analysis (PCA) with 95% variation is used, and the first two components are used for plot. Figure 5 shows seven groups. Then the K-means (Lloyd, 1982) cluster algorithm is performed with components extracted from PCA to group ROIs into seven parts. The comparison of DMN network for all algorithms is shown in Figure 6.

### 4.2. Case two

The second dataset has 46 subjects, including 15 men and 31 women. Their ages range from 44 to 65 years. The repetition time (TR) is 2 s; the number of slices is 64 and the time points are 175.

For this dataset, the fMRIPrep package (Esteban et al., 2017) is used for data preprocessing. The first ten time points are removed. The preprocessing pipeline and the procedure of data processing are analogous to the case-1.

**Figure 7:**
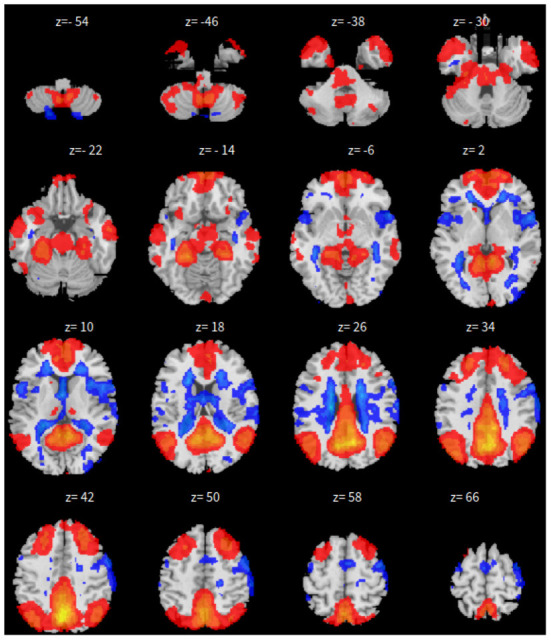
*xy*-plan view for SBC in Case-2.

**Figure 8:**
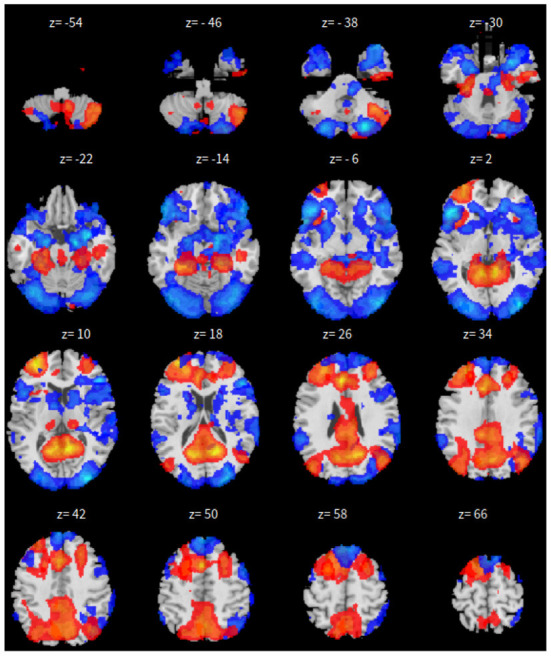
*xy*-plane view for ICA in Case-2.

**Figure 9:**
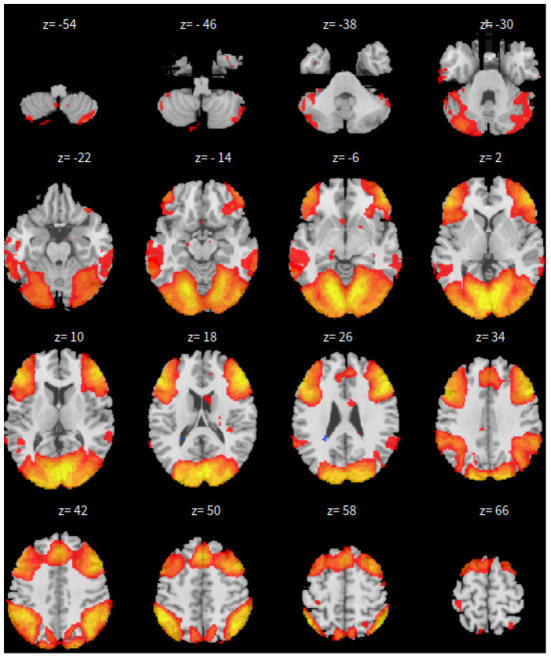
*xy*-plan view for DL in Case-2.

**Figure 10:**
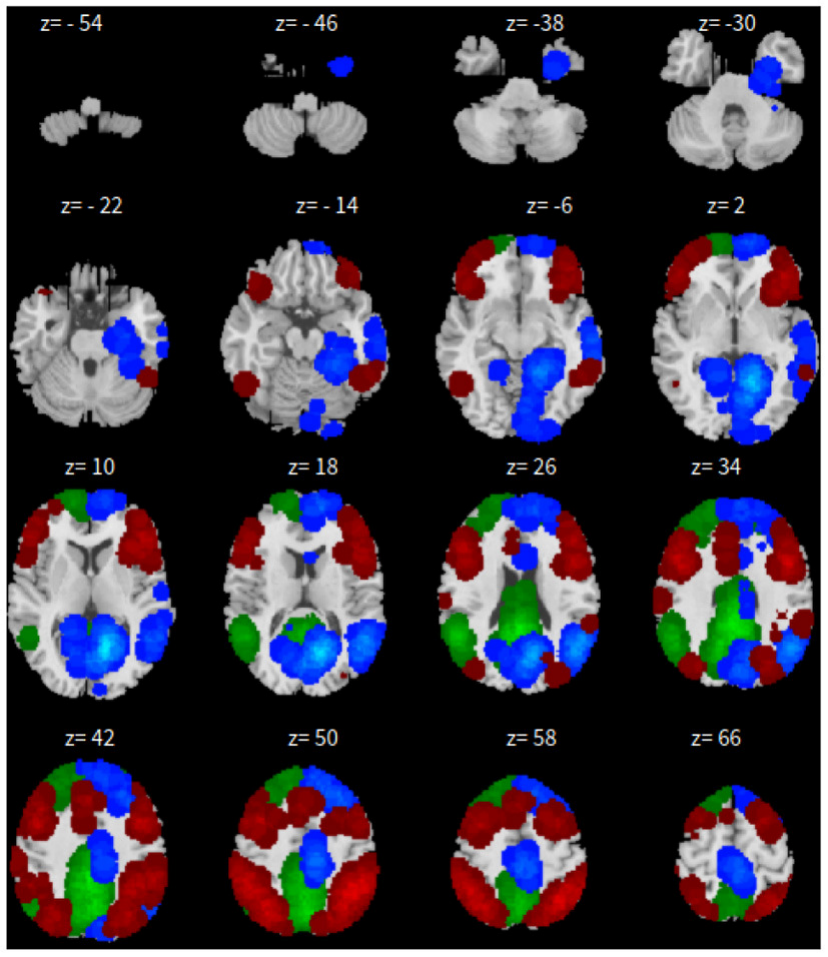
*xy*-plane view for GNN in Case-2.

**Figure 11:**
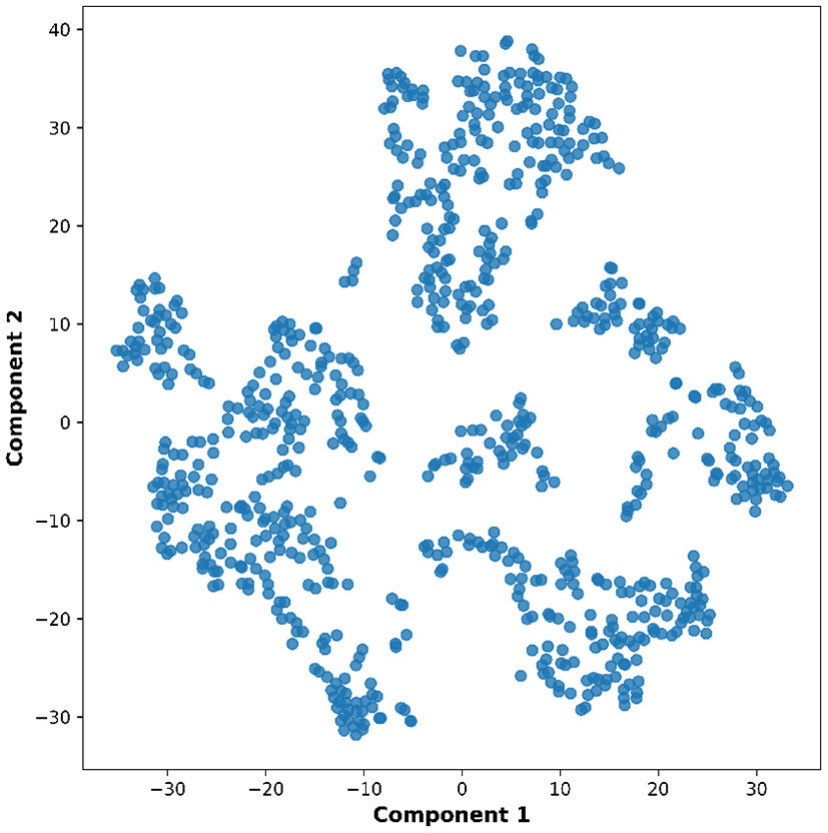
Plot of node embedding in Case-2 with two components.

**Figure 12:**
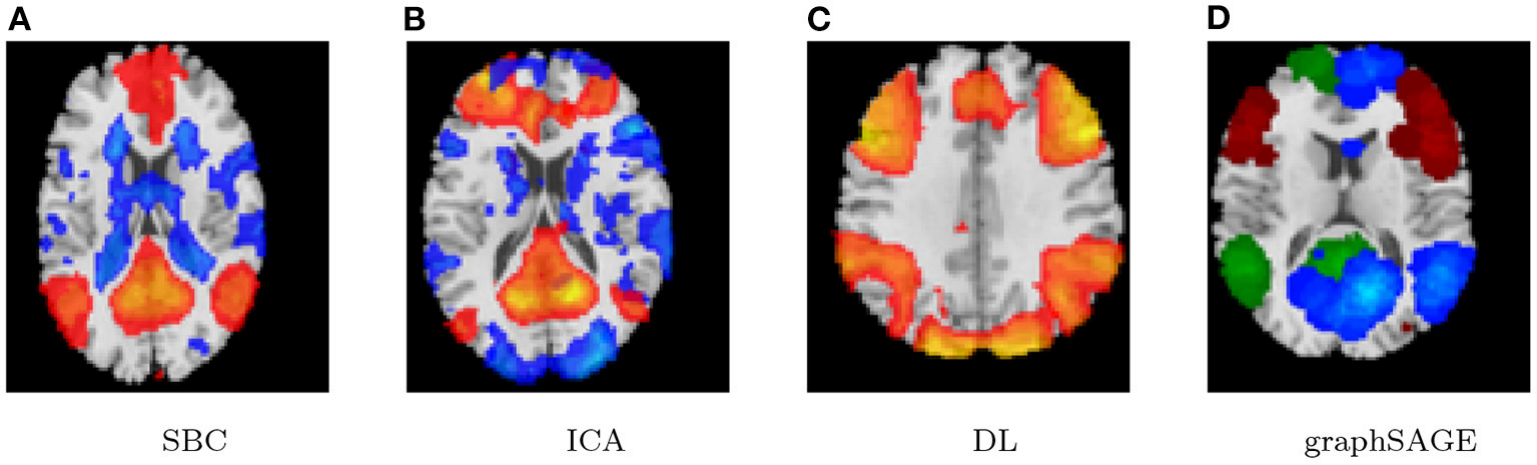
Case-2 DMN result comparison. **(A)** SBC, **(B)** ICA, **(C)**, and DL **(D)** graphSAGE.

The only difference is that, we only have 165 time points to compute the correlation between every pair of ROIs and 46 correlation matrices, corresponding to the 46 subjects. These are fed in to train the neural network. As all other parameters are set the same, the dropout rate is changed to 0.05 for this experiment. The results for SBC, fastICA, Dictionary Learning, and GraphSAGE, are shown in Figures 7–10, respectively. The 7-cluster node embedding based on GraphSAGE is shown in Figure 11. The comparison of DMN network from four algorithms is illustrated in Figure 12.

## 5. Discussion and conclusion

Noninvasive neuroimaging techniques such as fMRI have broadened and enhanced our understanding of the development and functions of the brain. The resting-state fMRI has gained popularity in studies of the brain's functional architecture and the assessment of neural networks of the brain since it measures integral resting-state functional connectivity across the whole brain without relying on any explicit tasks. It gives an ideal mean for examining possible changes of the whole brain network organization during the developments. Graph neural networks can be an effective framework for representation learning of functional connections. Farahani et al. (2019), provided a systematic review on applications of graph theory for identifying connectivity patterns in human brain networks. Machine learning on graph-structured network data has proliferated in a number of important applications. Graph-based network analysis reveals meaningful information of topological architecture of the brain networks and may provide novel insights into biological mechanisms underlying human cognition, health, and disease. Many graph neural networks achieve state-of-the-art results on nodes and graph classification tasks, but there is limited mathematical understanding of the GNN in general. Xu et al. (2019), presented a theoretical framework for analyzing the expressive power of GNN's to capture different graph structures.

As neuroimaging methods became more accurate, data continued to accumulate that suggested activity during resting states followed a certain pattern. The default mode network refers to this resting activity in the areas of the brain that are most active during these rest states (Raichle et al., 2001). The DMN research development showed that brain development is characterized by a trend of reduced segregation (i.e., local clustering) between spatially adjacent regions and increased integration of distant regions (Fair et al., 2007; Ma and Ma, 2018). Therefore, in regard to the study of DMN and FC for the brain, the knowledge on normal development of brain connectivity architecture could provide important insight into understanding the aspects of emergence, course, and severity of development-related brain disorders as well.

GraphSAGE is a framework for inductive representation learning on large graphs. GraphSAGE is used to generate low-dimensional vector representations for nodes, and is especially useful for graphs that have rich node attribute information. Oh et al. (2019), proposed a new data-driven sampling algorithm trained with reinforcement learning to replace the subsampling algorithm in graphSAGE. Transfer learning has proven successful for traditional deep learning problems. Kooverjee et al. (2022), recently demonstrated that transfer learning is effective with GNNs and compared the performances of graph convolution networks (GCN) and graphSAGE.

In this paper, we propose the use of a graph neural network to extract DMN-based functional connectivity on rs-fMRI data based on two data sets from the open shared neuroimage data resource (http://fcon_1000.projects.nitrc.org/fcpClassic/FcpTable.html).

As we can see in this study, the seed-based method can find the default mode network in two underlying cases and show a strong correlation with the prior selected seed of PCC. Its results are usually affected by the chosen seed and thus other possible networks are often ignored. The ICA and the dictionary learning methods can also find the default mode network in both cases, but these two methods provide some different networks relying on prior assumptions, some extracted networks are often difficult to interpret, and the results usually depend on the number of components.

The proposed graph neural network technique named graphSAGE can extract functional connectivity of DMN well based on the rs-fMRI data experiments. Furthermore, compared to the seed-based method, the ICA, as well as the DL, the graphSAGE method gives a more robust result. In addition, the three compared methods need to make prior threshold *p*-values for single subject analysis and group level analysis as well as to assume a certain number of components for ICA and dictionary learning before hand. Therefore, the analysis results often depend on these prior assumptions and are more or less subjective. In contrast, graphSAGE can be applied under relaxing restrictions and assumptions, as well as in consideration of the single subject analysis and group subjects analysis simultaneously. It can give more reliable results without subjective facts.

It is sometimes difficult to integrate all of the reported findings as pathological brain networks due to the fact that results often do not coincide with each other. Therefore, more consistent comparisons should be made across the studies. In addition, for some possible projects, statistical learning methods for longitudinal high-dimensional data (Chen et al., 2014) and longitudinal studies could be employed for monitoring brain network topological changes using different therapeutic strategies across a longer time duration (Mears and Pollard, 2016).

Many existing studies usually characterize static properties of the FC patterns, ignoring the time-varying dynamic information. A model of temporal-adaptive graph convolution network (TAGCN) to mine spatial and temporal information using rs-fMRI time series was studied in Yao et al. (2020). Textual descriptive information in the data can help improve classification and prediction in modeling. But one needs to use tools in natural language processing (NLP). For instance, in a recent work, Xu et al. (2022), used a newly developed NLP tool, called BERT to incorporate textual data in predictive modeling. Yao et al. (2019), used GCN for text classification in NLP. Incorporating textual data with GNN/GCN in fMRI data analysis could be a challenging but promising study.

## Data availability statement

Publicly available datasets were analyzed in this study. This data can be found here: 1000 Functional Connectomes Project (FCP), http://fcon_1000.projects.nitrc.org/fcpClassic/FcpTable.html.

## Ethics statement

Ethical review and approval was not required for the study on human participants in accordance with the local legislation and institutional requirements. Written informed consent from the patients/participants or patients/participants' legal guardian/next of kin was not required to participate in this study in accordance with the national legislation and the institutional requirements.

## Author contributions

DW carried out mainly the computing tasks and implemented the algorithm. All authors contributed equally to the design of the research, to the analysis of the results, and to the writing of the manuscript.

## Conflict of interest

The authors declare that the research was conducted in the absence of any commercial or financial relationships that could be construed as a potential conflict of interest.

## Publisher's note

All claims expressed in this article are solely those of the authors and do not necessarily represent those of their affiliated organizations, or those of the publisher, the editors and the reviewers. Any product that may be evaluated in this article, or claim that may be made by its manufacturer, is not guaranteed or endorsed by the publisher.
